# Association Between Adherence to the Mediterranean Diet and Metabolic Syndrome and Its Components Among Polish Postmenopausal Women: A Cross-Sectional Study

**DOI:** 10.3390/nu17172727

**Published:** 2025-08-22

**Authors:** Joanna Bajerska, Aleksandra Skoczek-Rubińska, Magdalena Dębińska-Kubiak, Wiktoria Stanisławska, Jarosław Walkowiak

**Affiliations:** 1 Department of Human Nutrition and Dietetics, Poznań University of Life Sciences, Wojska Polskiego 31, 60-624 Poznań, Poland; 2 Department of Dietetics, Faculty of Physical Culture, Poznań University of Physical Education, Estkowskiego 13, 66-400 Gorzów Wielkopolski, Poland; a.skoczek-rubinska@awf-gorzow.edu.pl; 3 Department of Pediatric Gastroenterology and Metabolic Diseases, Poznań University of Medical Sciences, Szpitalna 27/33, 60-572 Poznań, Poland

**Keywords:** adherence, Mediterranean diet, postmenopausal women, metabolic syndrome

## Abstract

**Background/Objectives**: The decrease in estrogen levels during menopause is associated with an increase in visceral fat accumulation, which can contribute to the development of metabolic syndrome (MetS). While some studies have suggested a link between the Mediterranean diet (MedDiet) and the reduced incidence of MetS and its components in the general population, these findings have not been confirmed among postmenopausal women. Therefore, this study investigated the association between the adherence to the MedDiet and the odds of having MetS, and established the food groups responsible for this effect in postmenopausal women. **Methods**: This cross-sectional study involved 312 postmenopausal women who underwent anthropometric measurements and blood parameter assessment. Adherence to the MedDiet was assessed using the Alternate Mediterranean Diet score (the aMED score), and MetS was defined based on the updated 2022 criteria. **Results**: After adjusting for potential confounders, adherence to the MedDiet was inversely associated with central obesity and hypertension. For each one-point increase in the aMED score (indicating a better adherence to the MedDet), the odds of central obesity and hypertension were significantly reduced by 33% (OR = 0.669, 95% CI: 0.518; 0.866, *p* = 0.002) and by 18% (OR = 0.817, 95% CI: 0.689; 0.969, *p* = 0.020), respectively. A greater consumption of nuts and fish was associated with lower odds of central obesity (OR = 0.972, 95% CI: 0.950; 0.995; *p* = 0.016) and (OR = 0.989, 95%CI: 0.979; 1.000; *p* = 0.043), respectively, whereas high processed red meat consumption was associated with hypertension (OR = 1.004, 95% CI: 1.000; 1.008, *p* = 0.048). **Conclusions**: A greater adherence to the MedDiet was associated with lower odds of central obesity and hypertension among postmenopausal women; however, it did not translate to a reduced likelihood of having MetS. A higher consumption of nuts and fish was associated with lower odds of central obesity, whereas a higher consumption of red, processed meats was associated with higher odds of hypertension. Longitudinal studies are needed to determine the causality of these relationships.

## 1. Introduction

The number of women entering menopause, typically aged 45–54, in the coming decades is set to increase significantly, with 1.65 billion projected by 2050 [1]. From a physiological point of view, menopause is the cessation of ovarian function, with the loss of reproductive hormone production and irreversible loss of fertility [2]. Hormone changes in menopause affect lipid metabolism, energy consumption, insulin resistance, and body fat composition, with a transition from a gynecoid to an android body shape and increased abdominal and visceral fat accumulation associated with increased risks of metabolic syndrome (MetS) [2]. The new definition of MetS proposed by Dobrowolski et al., 2022 encompasses the presence of obesity (abdominal obesity or obesity prescribed by BMI) and two of the three following criteria: hypertension, impaired glucose metabolism, and elevated non-HDL cholesterol [3]. The prevalence of MetS in postmenopausal women is significantly higher than in premenopausal women, with reported prevalence rates ranging from 32 to 58% [4].

While numerous traditional dietary patterns play an important role in preventing and treating MetS and its constituents, the Mediterranean diet (MedDiet) is one of the most extensively researched. For example, Di Daniele et al. suggested in their review that the MedDiet can be used as a possible therapy for MetS, as it prevents excesses of adiposity and the obesity-related inflammatory response [5]. A meta-analysis of twelve cross-sectional and prospective cohorts showed that a higher adherence to the MedDiet was associated with a 19% lower risk of developing MetS [relative risk (RR): 0.81 (95% confidence interval (CI) 0.71 to 0.92]; its individual components, such as waist circumference and blood pressure, also became less likely [RR: 0.82 (95% CI 0.70 to 0.96); RR: 0.87 (95% CI 0.77 to 0.97), respectively [6]. However, adherence to the MedDiet in the sub-analysis, which included only women (two studies), was not significantly associated with a reduced risk of MetS [6]. Moreover, among the studies included in the meta-analysis, those conducted in non-Mediterranean countries reported no significant effects [6]. Leone et al. (2022) observed that a greater adherence to the MedDiet reduced the risk of metabolically unhealthy obesity in postmenopausal, but not in premenopausal, women living in Italy [7]. While the MedDiet can be beneficial for overall health, including cardiovascular health, there is little strong evidence that specifically links the MedDiet to a reduced incidence of MetS among postmenopausal women, especially those living far away from the Mediterranean region. Therefore, this study aimed to investigate the association between adherence to the MedDiet and the odds of having MetS and its components among Polish postmenopausal women, and to establish the food groups responsible for this effect.

## 2. Materials and Methods

### 2.1. Subjects

**Figure 1:**
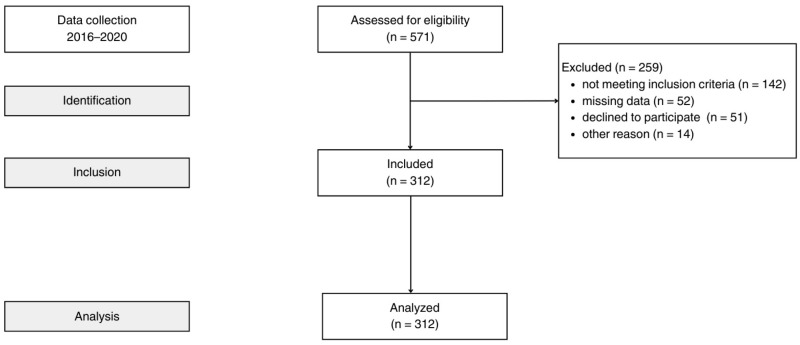
STROBE flow chart.

This cohort study recruited Polish postmenopausal women between 2016 and 2020 who had an absence of menses during the 12 months before the study. The exclusion criteria were as follows: unnatural menopause (e.g., due to surgery or radiation therapy for cervical cancer), use of hormonal replacement therapy, a history of heart disease, insulin-dependent diabetes, type-2 diabetes, hypothyreosis, chronic inflammatory disease, liver disease, or any type of cancer. The analysis included 312 menopausal women (Figure 1).

This study was approved by the Bioethics Committee of Poznan University of Medical Sciences (number 664/20 and 603/14), and was conducted as per the Declaration of Helsinki. All subjects provided written informed consent before study participation.

### 2.2. Metabolic Syndrome Definition

The diagnostic criteria for MetS are based on the guidelines published by Dobrowolski et al. [3] which take into account the presence of obesity, either a waist circumference in women ≥ 88 cm or BMI ≥ 30 kg/m^2^, and two of the following criteria: hypertension (systolic blood pressure ≥ 130, diastolic blood pressure ≥ 85 mm Hg), or antihypertensive treatment, impaired glucose metabolism (fasting glucose ≥ 100 mg/dL) or glucose lowering drug treatment, non-HDL cholesterol level ≥ 130 mg/dL, or lipid-lowering drug treatment.

### 2.3. Dietary Assessment

A three-day food diary was used to assess dietary intake, allowing respondents to record all foods and beverages consumed over a specific period of two weekdays and one weekend day. A dietitian provided twenty minutes of training to improve the accuracy of the food records. This training involved face-to-face education on how to accurately record real-time food intake, including the name of the food, amount of food consumed using standard household measurements, preparation methods, brand names of commercially available products, and recipes of composite dishes. Instructions on how to use paper-based or web-based (http://www.ilewazy.pl/) photographs of products and dishes were also provided, and the respondents were provided with comprehensive written instructions on how to collect dietary data. The dietitian reviewed the completed records with the respondent to clarify potential omissions and ambiguities or make necessary changes. The reported food and beverage quantities were converted into grams and milliliters, respectively, and then input into dietary analysis software Dieta 6.0 (National Food and Nutrition Institute, Warsaw, Poland) to calculate the energy and nutritional value of their daily food intake.

### 2.4. Anthropometry

The anthropometric data recorded included body weight (kg), height (cm), body mass index (BMI = weight/height^2^), fat mass (%), fat-free mass (kg), waist circumference (cm), and waist-to-height ratio (WHtR = waist circumference/height). Height was measured to the nearest 0.1 cm using a stadiometer (RadWag, Radom, Poland). Body weight was assessed to the nearest 0.1 kg after an overnight fast using the calibrated scale. Body composition was measured using dual-energy X-ray absorptiometry (DXA) with a Lunar Prodigy densitometer (GE Healthcare, Madison, WI, USA, 2013). Waist circumference (WC) was measured using nonelastic tape placed horizontally above the iliac crest with minimal respiration. The cutoff used for WC was that for women of European ethnicity (≥88 cm) [8]. Systolic and diastolic blood pressure were measured using a sphygmomanometer.

### 2.5. Biochemistry

Blood was drawn from all participants by qualified personnel in the morning after an overnight fast, and the serum was obtained by centrifugation at 1000 rpm for 10 min. The biological material was stored at −80 °C until further analysis. Biochemical parameters, including glucose (GLU), total cholesterol (T-C), high-density lipoprotein cholesterol HDL, and triglycerides (TG), were determined using a Beckman Coulter AU analyzer (Beckman Coulter, Inc., Brea, CA, USA). Low-density lipoprotein cholesterol (LDL) concentrations were calculated using the Friedewald formula, and non-HDL cholesterol was calculated by subtracting HDL-C from total cholesterol.

### 2.6. Physical Activity Assessment

Physical activity was assessed using a short version of the International Physical Activity Questionnaire and was classified as low (≤600 metabolic equivalent [MET]/min/week), moderate (600–1499 MET/min/week), and high (≥1500 MET/min/week).

### 2.7. Mediterranean Diet Adherence

Adherence to the MedDiet was determined using the alternate Mediterranean Diet (aMED) score, originally described by Fung et al. [9]. Briefly, the aMED includes nine components: vegetables, legumes, fruits, nuts, whole grains, red and processed meats, fish, alcohol, and the ratio of monounsaturated to saturated fats. Median cut-points were calculated for all components, with one point awarded if the individual scored above the median for intake of vegetables, fruits, legumes, whole grain, nut, and fish intake, or if the monounsaturated:saturated fat ratio was above the median; one point was also awarded if the individual consumed median of less intake of red and processed meats. One point was given for consumption of 5–15 g of ethanol per day for women, which represents approximately 300 mL of regular beer, 150 mL of wine, or 35 mL of liquor, and zero points were awarded for consumption outside of these ranges. The total score ranged from zero to nine points, with higher scores indicating greater adherence to the MedDiet.

### 2.8. Statistical Analysis

Statistical analysis was performed using v. 13.0 Statistica software (TIBCO Software, Palo Alto, CA, USA). The Shapiro–Wilk test was used to verify whether a dataset follows a normal distribution. Since most variables did not meet the assumption of normality, non-parametric tests were used. Participants were grouped into tertiles based on aMED scores: tertile 1 (scores ≤ 3) represented those with the weakest adherence to the Mediterranean diet, whereas tertile 3 (scores ≥ 5) included those with the strongest adherence. Continuous variables are presented as medians with interquartile ranges (IQR), and categorical variables as counts and percentages (*n*, %). The Kruskal–Wallis test and Pearson’s chi-square test were applied to compare continuous and categorical variables across aMED tertiles, respectively.

Unadjusted and adjusted logistic regression analyses were conducted to examine the association between adherence to the Mediterranean diet (aMED score) and the odds of metabolic syndrome (MetS) and its individual components. The dependent variables were the presence of MetS and each of its components (dichotomous: 0 = no, 1 = yes), and the aMED score was treated as a continuous predictor. Covariates included total energy intake, years of education, years since menopause, physical activity level, and smoking status.

Additionally, unadjusted and adjusted logistic regression models were used to assess which individual food items typical of the Mediterranean diet were associated with the odds of MetS and its components. In these models, the dependent variables remained the same, while the consumption of specific food items (continuous) was used as the main predictor. The same set of covariates was included. A *p*-value < 0.05 (two sided) was considered statistically significant.

## 3. Results

The analysis included 312 menopausal women with a median age of 58 years (IQR: 53.0–63.0 years old) and a median aMED score of 4.0 (IQR: 3.0–5.0). Participants with a higher adherence to the MedDiet (tertile 3) generally exhibited a healthier diet, including a higher intake of vegetables, fruits, nuts, whole grains, legumes, and fish, while consuming less red and processed meats. Moreover, significantly more postmenopausal women with a higher adherence to the MedDiet stated that they consumed 5–15 g of ethanol in alcoholic drinks per day (Table 1). Significant differences were only observed in some of the MetS components, including the BMI, systolic blood pressure, and HDL-C levels. A higher aMED score was associated with a lower prevalence of central obesity (74% vs. 92%), hypertension (57% vs. 75%), and MetS (50% vs. 70%) in this study population (Table 1).

Table 2 shows the association between adherence to the MedDiet and the odds of MetS and its components. In the crude model, there was an association between adherence to the MedDiet and the odds of obesity, central obesity, hypertension, and MetS. However, after adjusting for relevant confounders, adherence to the MedDiet was associated only with lower odds of having central obesity and hypertension, although the prevalence of obesity and MetS in our population was as high as 52% and 61%, respectively (Table 1). In particular, for each one-point increase in the aMED score (indicating a better adherence to the MedDiet), the odds of central obesity and hypertension significantly reduced by 33% (OR = 0.669, 95% CI: 0.518; 0.866, *p* = 0.002) and by 18% (OR = 0.817, 95% CI: 0.689; 0.969, *p* = 0.020), respectively (Table 2).

In the fully adjusted model of how individual food items related to the odds of central obesity and hypertension (Table 3), a higher consumption of nuts and fish was associated with a lower odds of central obesity (OR = 0.972, 95% CI: 0.950; 0.995; *p* = 0.016) and (OR = 0.989, 95% CI: 0.979; 1.000; *p* = 0.043), respectively, whereas a higher consumption of red processed meats was associated with a greater odds of hypertension (OR = 1.004, 95% CI: 1.000; 1.008, *p* = 0.048). There were no associations between the incidence of central obesity and hypertension and the consumption of other food items, such as vegetables, legumes, fruit, whole grains, the monounsaturated to saturated fat ratio, or alcohol intake.

## 4. Discussion

This cross-sectional study was designed to investigate the association between adherence to the Mediterranean diet and the odds of having MetS and its components among Polish postmenopausal women. Based on the findings from our study, a greater adherence to the MedDiet was associated with lower odds of central obesity and hypertension among postmenopausal women, but it did not translate to a reduced likelihood of having MetS. More specifically, for each one-point increase in the aMED score (indicating a better adherence to the MedDiet), the odds of having central obesity and hypertension were significantly reduced by 33% and 18%, respectively.

The food groups associated with lower odds of central obesity were nuts and fish, whereas a higher consumption of processed red meat was associated with hypertension. To the best of our knowledge, this is the first complex study focused on this target population. Previously, Leone et al. reported that a greater adherence to the MedDiet reduced the risk of metabolically unhealthy obesity in postmenopausal but not in premenopausal women [7]. Overall, our findings regarding individual metabolic alterations are in line with those of previous articles, where adherence to the MedDiet is inversely associated with abdominal adiposity [10] and hypertension [11]. The beneficial effect of this diet in reducing central adiposity may be associated with its high content of polyunsaturated fatty acids (PUFAs) and MUFAs and low levels of saturated fatty acids (SFAs) [12]. Visceral adipose tissue consists predominantly of SFAs, whereas subcutaneous fat has deposits of PUFAs and MUFAs [13]. The main sources of *n*-3 PUFAs in the MedDiet are fish, seafood, and nuts, while olive oil is the main source of MUFAs [14]. Indeed, in our study, the food groups most significantly associated with lower odds of central obesity in postmenopausal women were nuts and fish. Specifically, the most prominent fatty acids in nuts are linoleic acid, oleic acid, and α-linolenic acid, the latter being especially abundant in walnuts [15]. Cubas-Basterrechea et al. observed that the consumption of less than the recommended 30 g of nuts at least three times a week was linked to a 19% higher prevalence of central obesity and a 61% higher prevalence of MetS compared to the recommended level in an older Spanish population [16]. A dose–response meta-analysis of prospective observational studies found an inverse association between nut consumption and abdominal obesity (RR: 0.42, 95% CI: 0.31, 0.57). However, the authors also indicated that the quality of the evidence was rather low [17]. Several studies have explored the effects of fish consumption on the risk of developing MetS. In the SEAFOODPlus study, in which 126 overweight adults were randomized to a calorie-restricted diet with or without 150 g/day of fish (cod), five times per week for eight weeks, participants who consumed fish lost 1.7 kg more body weight than the control group, and experienced a 3.4 cm reduction in WC and a 5.2 mmHg reduction in systolic blood pressure [18]. In a South Korean cohort study, an average daily fish consumption of between 40 and 70 g per day was associated with a 57% reduction in the risk of developing MetS, albeit only among men [19]. An earlier dose–response meta-analysis of prospective observational studies also found fish intake to be associated with reduced abdominal obesity [17].

In our study, processed red meat consumption was associated with higher odds of hypertension, consistent with findings from previous cross-sectional studies [20,21]. However, an increased risk of developing hypertension in a Brazilian population was associated with a moderate and high consumption of processed meats but not with the consumption of red meat [22]. It is of note that the available data also demonstrate that the intake of white meat (such as poultry) is associated with more favorable hypertension outcomes than the consumption of red meat [23]. Many hypotheses exist regarding the association of red meat with a higher risk of hypertension, particularly that of processed red meat. The higher risk associated with processed red meat may be related to its sodium content, additives, and their metabolism by the gut microbiome into deleterious metabolites [23]. Studies to date have outlined the potential mechanistic roles of SCFAs in mediating host–microbe communication in hypertension, often acting via host SCFA G-protein-coupled receptors. Similarly, there are clear roles for trimethylamine-N oxide (TMAO) in cardiovascular diseases [24].

Although the cross-sectional study conducted in 10 European countries, found an association between adherence to the Mediterranean diet (rich in plant-based foods and unsaturated fatty acids) and lower abdominal adiposity in men and women [25], we did not observe such an association with the plant-based components of the MedDiet (e.g., fruits and vegetables, olive oil, and legumes). The lack of an observed association may be attributed to methodological limitations, as focusing on individual food items does not take into account other dietary factors that may influence the results, nor the synergistic effects between foods and nutrients [26]. Although a recent meta-analysis of observational studies provided evidence that a greater adherence to the MedDiet was associated with reduced odds of having MetS [6], no such association was found in the present study, especially after adjusting for the relevant confounders. This suggests that the observed association was likely due to confounding variables rather than a direct causal link between the MedDiet and MetS. Indeed, it was suggested that women, non-smokers, and physically active with a higher educational level have a healthier dietary pattern [27]. In our study population, no associations were found also between adherence to the MedDiet and the odds of having hyperglycemia or elevated non-HDL cholesterol levels. Papadaki et al. (2020) in a meta-analysis of RCTs observed a greater effect of the MedDiet on blood glucose but only in studies conducted in Mediterranean countries compared to non-Mediterranean countries [28]. This may be due to the better availability of the required food items, and other eating-related behaviors such as the time of eating, the order of courses in each meal, or sun exposure in the Mediterranean region [29]. Indeed, Boujelbane et al., 2025 noted that those living in Mediterranean regions show a better adherence to traditional MedDiet components (legumes and fish) than those from non-Mediterranean regions [30]. These findings align with those of previous research showing that Mediterranean populations are more likely to adhere to the principles of the MedDiet [31]. Previously, we also showed the better effects of the Central European Diet in a group of postmenopausal women, possibly because of the native character of this diet for the target population [32,33].

The present study findings should be considered in light of some limitations. First, our study has an observational design, and cannot establish causal relationships between adherence to the MedDiet and MetS or its components, only associations. Second, as with all studies that use self-reported dietary assessment methods, the widely acknowledged underreporting of energy intake, especially among women with a higher BMI, may cause selection bias. For this reason, energy intake was used as a potential confounder. Moreover, the short time frame may not accurately represent an individual’s typical dietary intake, due to the daily variations in food and nutrient consumption. Third, only postmenopausal women were included; therefore, these findings cannot be generalized to other populations. Moreover, being diagnosed with MetS could lead to behavior changes in dietary intake, which could represent another potential limitation. A final limitation of the study is the omission of potential confounders, such as medication use, which may have influenced the observed associations.

Nonetheless, this study has several strengths, especially considering that only postmenopausal women were included in the study. Our findings that the Mediterranean dietary pattern may help prevent the development of central obesity and hypertension in postmenopausal women are, therefore, very strong. Moreover, the use of the aMED score as a tool for evaluating adherence to the MedDiet may be used even in populations outside the Mediterranean region [34].

## 5. Conclusions

Based on the findings from our study, a greater adherence to the MedDiet was associated with lower odds of having abdominal obesity and hypertension in a group of postmenopausal women, but it did not translate into a reduction in the odds of having MetS. For each one-point increase in the aMED score (indicating a better adherence to the MedDiet), the odds of having central obesity and hypertension were significantly reduced by 33% and 18%, respectively. The higher consumption of nuts and fish was associated with lower odds of central obesity, whereas a higher consumption of red, processed meats was associated with higher odds of hypertension. Longitudinal studies are needed in order to determine the causality of these relationships. It is recommended that adherence to the MedDiet be encouraged in non-Mediterranean populations. This should focus on education, cultural adaptation, and addressing practical barriers, highlighting the health benefits of the Mediterranean diet, and providing support through nutritional guidance and potentially by subsidizing access to key dietary components.

## Figures and Tables

**Table 1:** Characteristics of the study participants.

	Participants by Mediterranean Diet Adherence Score				
Variables	All (*n* = 312)	T1: 0–3 (*n* = 149)	T2: 4 (*n* = 74)	T3: 5–9 (*n* = 90)	*p*-value *
	Median (1st–3rd quartile)	Median (1st–3rd quartile)	Median (1st–3rd quartile)	Median (1st–3rd quartile)	
	**Sociodemographic and Anthropometric Data**				
Age [y]	58.0 (53.0–63.0)	58.0 (53.0–62.0)	58.0 (54.0–63.0)	57.0 (52.0–62.0)	0.594
Years since menopause [y]	6.0 (2.0–11.0)	7.0 (2.0–11.0)	6.0 (2.0–12.0)	5.0 (2.0–11.0)	0.616
Duration of education [y]	17.0 (17.0–17.0)	12.0 (12.0–17.0)	17.0 (12.0–17.0)	17.0 (17.0–17.0)	<0.001
Current or former smoker (*n*, %)	64 (20.5)	38 (26)	13 (18)	38 (14)	0.099
Body weight [kg]	78.6 (68.2–88.5)	79.7 (72.4–88.6)	79.8 (70.7–88.6)	73.9 (63.6–87.0)	0.057
FAT [kg]	36.0 (27.6–44.2)	37.0 (30.8–44.3)	36.4 (28.5–44.0)	32.7 (24.0–44.0)	0.100
FFM [%]	54.3 (49.2–59.3)	53.7 (48.9–58.3)	54.5 (49.8–59.9)	54.9 (49.5–61.6)	0.188
WHtR	0.62 (0.58–0.68)	0.63 (0.59–0.68)	0.62 (0.58–0.68)	0.62 (0.54–0.68)	0.100
	**Dietary Intake**				
Vegetables [g/d]	318.8 (202.0–452.4)	232.5 (158.4–361.8)	378.2 (259.7–453.6)	402.2 (321.4–520.6)	<0.001
Legumes [g/d]	0.0 (0.0–0.0)	0.0 (0.0–0.0)	0.0 (0.0–0.0)	0.0 (0.0–10.0)	<0.001
Fruit [g/d]	224.6 (109.2–378.8)	160.7 (81.3–269.2)	271.9 (132.5–440.3)	322.2 (184.8–423.0)	<0.001
Nuts [g/d]	0.0 (0.0–4.1)	0.0 (0.0–0.0)	0.0 (0.0–4.0)	2.5 (0.0–10.0)	<0.001
Whole grains [g/d]	76.3 (36.5–121.7)	53.3 (17.9–110.0)	86.3 (47.0–121.6)	100.0 (63.3–136.7)	<0.001
Red and processed meats [g/d]	60.2 (28.6–107.6)	75.0 (41.9–121.5)	54.3 (30.0–96.6)	38.0 (12.8–77.5)	<0.001
Fish [g/d]	0.0 (0.0–30.0)	0.0 (0.0–0.0)	0.0 (0.0–36.7)	24.5 (0.0–50.0)	<0.001
MUFA:SFA	1.0 (0.82–1.22)	0.98 (0.82–1.26)	0.99 (0.82–1.18)	1.1 (0.84–1.26)	0.302
Alcohol intake [g/d]	0.0 (0.0–2.8)	0.0 (0.0–0.0)	0.0 (0.0–3.9)	0.0 (0.0–5.6)	0.004
Alcohol intake 5–15 g/day (*n*, %)	46 (15)	10 (7)	12 (16)	24 (27)	<0.001
Energy intake [kcal/d]	1596.8 (1311.7–1946.3)	1544.9 (1268.2–1904.5)	1498.5 (1291.7–1953.8)	1731.5 (1453.8–2015.2)	0.035
%E protein	16.4 (14.0–18.8)	16.7 (14.4–19.1)	15.9 (13.6–18.8)	15.6 (13.6–18.5)	0.125
%E fat	22.9 (12.9–32.8)	22.0 (13.8–33.2)	19.0 (11.2–30.0)	27.5 (13.5–32.9)	0.088
%E carbohydrate	53.3 (45.8–60.4)	51.7 (44.8–59.7)	53.0 (48.4–63.6	52.1 (46.4–58.6)	0.165
Dietary fiber [g/d]	21.4 (15.9–26.8)	18.3 (13.3–23.7)	22.9 (17.2–28.1)	24.6 (19.9–30.1)	<0.001
	**Physical Activity**				
Low < 600 MET/min/wk (*n*, %)	64 (20.5)	28 (19)	21 (28)	15 (17)	0.148
Moderate 600–1499 MET/min/wk (*n*, %)	222 (71)	112 (75)	45 (61)	65 (72)	
High > 1499 MET/min/wk (*n*, %)	26 (8.0)	9 (6)	8 (11)	10 (11)	
	**MetS and Its Components**				
BMI [kg/m^2^]	30.3 (26.2–34.5)	30.7 (27.6–34.6)	30.4 (26.3–34.0)	28.5 (23.2–34.9)	0.024
***Obesity ≥ 30 kg/m^2^ (n, %)***	***162 (52)***	***84 (56)***	***39 (93)***	***40 (44)***	***0.196***
Waist circumference [cm]	101.0 (93.8–109.2)	102.0 (96.0–109.0)	101.0 (94.5–109.0)	99.3 (87.5–110.0)	0.209
***Central obesity ≥ 88 cm (n, %)***	***273 (76)***	***137 (92)***	***69 (95)***	***67 (74)***	***<0.001***
Systolic blood pressure [mmHg]	133.5 (124.7–147.0)	134.0 (129.7–147.0)	137.0 (121–154.0)	130.0 (122–140.0)	0.118
Diastolic blood pressure [mmHg]	83.8 (79.0–92.0)	84.0 (79.7–91.0)	86.0 (78.3–94.7)	81.5 (79.0–90.0)	0.319
***Hypertension ≥ 130/85 mmHg (n, %)***	***208 (66.7)***	***112 (75)***	***45 (62)***	***51 (57)***	***0.008***
Blood glucose [mg/dL]	94.7 (86.0–100.8)	94.1 (87.0–102.0)	94.9 (86.0–100.0)	94.5 (85.0–100.0)	0.830
***Blood glucose ≥ 100 mg/dL (n, %)***	***87 (28)***	***47 (33)***	***18 (26)***	***23 (27)***	***0.431***
Triglycerides [mg/dL]	126.0 (86.8–170.0)	128.3 (91.0–167.6)	125.5 (89.0–167.0)	118.9 (79.0–178.3)	0.447
Total Cholesterol [mg/dL]	227.0 (198.2–255.0)	226.0 (201.0–252.0)	227.5 (196.3–253.3)	227.5 (196.0–260.8)	0.995
HDL-C [mg/dL]	58.0 (48.0–68.0)	56.6 (47.0–66.0)	60.5 (49.9–70.0)	58.0 (50.0–69.0)	0.256
non-HDL [mg/dL]	166.5 (136.0–196.0)	169.2 (140.0–198.0)	161.0 (135.0–195.7)	160.4 (134.0–195.0)	0.734
***Non-HDL ≥ 130 mg/dL (n, %)***	***243 (78)***	***117 (82)***	***57 (81)***	***69 (80)***	***0.919***
LDL-C [mg/dL]	139.0 (114.0–162.9)	139.0 (115.3–165.0)	137.5 (112.0–159.4)	141.0 (110.0–162.9)	0.899
***Metabolic syndrome (yes, %)***	***191 (61)***	***104 (70)***	***42 (58)***	***45 (50)***	***0.007***

The results are presented as medians with IQR. Categorical data as *n* (%). * The categorical data were analyzed using Chi-squared test, while Kruskal–Wallis test was applied to continuous data. Abbreviations: BMI: body mass index (calculated as weight in kilograms divided by height in meters squared); HDL: high-density lipoprotein; MUFA:SFA: monounsaturated fatty acids to saturated fatty acids; LDL: low-density lipoprotein; T: tertile; WHtR: waist-to-height ratio (calculated as waist circumference divided by height in centimeters).

**Table 2:** Association between adherence to the MedDiet and odds of having MetS and its components.

Variables	OR	95% CI	*p*-Value
**Obesity ≥ 30 kg/m^2^**			
Crude model	0.848	0.730; 0.986	0.032
Adjusted model	0.904	0.760; 1.074	0.250
**Central obesity ≥ 88 cm**			
Crude model	0.633	0.500; 0.803	<0.001
Adjusted model	0.669	0.518; 0.866	0.002
**Hypertension ≥ 130/85 mmHg**			
Crude model	0.779	0.663; 0.915	0.002
Adjusted model	0.817	0.689; 0.969	0.020
**Non-HDL-C levels ≥ 130 mg/dL**			
Crude model	1.080	0.887; 1.316	0.443
Adjusted model	1.086	0.879; 1.342	0.444
**Blood glucose ≥ 100 mg/dL**			
Crude model	0.851	0.717; 1.009	0.064
Adjusted model	0.875	0.726; 1.055	0.163
**Presence of MetS (yes)**			
Crude model	0.810	0.693; 0.945	0.008
Adjusted model	0.863	0.730; 1.019	0.082

Values are odds ratios (OR) and 95% CI obtained from logistic regression models. The multivariate model was adjusted for energy intake and years of education, years since menopause, category of physical activity, and smoking status.

**Table 3:** Association of individual food items with the odds of central obesity and hypertension.

Variables	Central Obesity			Hypertension		
	OR	95% CI	*p*-Value	OR	95% CI	*p*-Value
Vegetables [g/d]	0.998	0.996; 1.000	0.059	0.999	0.998; 1.000	0.137
Legumes [g/d]	1.011	0.979; 1.045	0.503	1.002	0.989; 1.016	0.725
Fruit [g/d]	0.999	0.998; 1.001	0.537	0.999	0.998; 1.001	0.290
Nuts [g/d]	0.972	0.950; 0.995	0.016	0.994	0.975; 1.014	0.558
Whole grains [g/d]	0.996	0.991; 1.002	0.203	1.003	0.999; 1.007	0.194
Red and processed meats [g/d]	1.004	0.998; 1.011	0.192	1.004	1.000; 1.008	0.048
Fish [g/d]	0.989	0.979; 1.000	0.043	0.995	0.987; 1.003	0.186
MUFA:SFA	1.070	0.314; 3.644	0.914	1.315	0.593; 2.919	0.500
Alcohol intake [g/d]	1.004	0.950; 1.061	0.891	0.994	0.954; 1.036	0.771

Values are odds ratios (OR) and 95% CI obtained from logistic regression models adjusted for energy intake and years of education, years since menopause, category of physical activity, and smoking status. MUFA:SFA: monounsaturated fatty acids to saturated fatty acids.

## Data Availability

The data presented in this study are available upon request from the corresponding author due to privacy and ethical restrictions.

## References

[1] Lau B.H.P., Tang C.S.K., Holroyd E., Wong W.C.W. (2024). Challenges and Implications for Menopausal Health and Help-Seeking Behaviors in Midlife Women from the United States and China in Light of the COVID-19 Pandemic: Web-Based Panel Surveys. JMIR Public Health Surveill..

[2] Davis S.R., Pinkerton J., Santoro N., Simoncini T. (2023). Menopause—Biology, Consequences, Supportive Care, and Therapeutic Options. Cell.

[3] Dobrowolski P., Prejbisz A., Kuryłowicz A., Baska A., Burchardt P., Chlebus K., Dzida G., Jankowski P., Jaroszewicz J., Jaworski P. (2022). Metabolic Syndrome—A New Definition and Management Guidelines. Arter. Hypertens..

[4] Jeong H.G., Park H. (2022). Metabolic Disorders in Menopause. Metabolites.

[5] Di Daniele N., Noce A., Vidiri M.F., Moriconi E., Marrone G., Annicchiarico-Petruzzelli M., D’Urso G., Tesauro M., Rovella V., De Lorenzo A. (2017). Impact of Mediterranean Diet on Metabolic Syndrome, Cancer and Longevity. Oncotarget.

[6] Godos J., Zappalà G., Bernardini S., Giambini I., Bes-Rastrollo M., Martinez-Gonzalez M. (2017). Adherence to the Mediterranean Diet Is Inversely Associated with Metabolic Syndrome Occurrence: A Meta-Analysis of Observational Studies. Int. J. Food Sci. Nutr..

[7] Leone A., De Amicis R., Battezzati A., Bertoli S. (2022). Adherence to the Mediterranean Diet and Risk of Metabolically Unhealthy Obesity in Women: A Cross-Sectional Study. Front. Nutr..

[8] Alberti K.G.M.M., Eckel R.H., Grundy S.M., Zimmet P.Z., Cleeman J.I., Donato K.A., Fruchart J.-C., James W.P.T., Loria C.M., Smith S.C. (2009). Harmonizing the Metabolic Syndrome: A Joint Interim Statement of the International Diabetes Federation Task Force on Epidemiology and Prevention; National Heart, Lung, and Blood Institute; American Heart Association; World Heart Federation; International Atherosclerosis Society; and International Association for the Study of Obesity. Circulation.

[9] Fung T.T., Rexrode K.M., Mantzoros C.S., Manson J.E., Willett W.C., Hu F.B. (2009). Mediterranean Diet and Incidence of and Mortality from Coronary Heart Disease and Stroke in Women. Circulation.

[10] Muscogiuri G., Verde L., Sulu C., Katsiki N., Hassapidou M., Frias-Toral E., Cucalón G., Pazderska A., Yumuk V.D., Colao A. (2022). Mediterranean Diet and Obesity-Related Disorders: What Is the Evidence?. Curr. Obes. Rep..

[11] Nissensohn M., Román-Viñas B., Sánchez-Villegas A., Piscopo S., Serra-Majem L. (2016). The Effect of the Mediterranean Diet on Hypertension: A Systematic Review and Meta-Analysis. J. Nutr. Educ. Behav..

[12] Marventano S., Kolacz P., Castellano S., Galvano F., Buscemi S., Mistretta A., Grosso G. (2015). A Review of Recent Evidence in Human Studies of *N*-3 and *n*-6 PUFA Intake on Cardiovascular Disease, Cancer, and Depressive Disorders: Does the Ratio Really Matter?. Int. J. Food Sci. Nutr..

[13] Calder P.C., Harvey D.J., Pond C.M., Newsholme E.A. (1992). Site-specific Differences in the Fatty Acid Composition of Human Adipose Tissue. Lipids.

[14] Mantzioris E., Muhlhausler B.S., Villani A. (2022). Impact of the Mediterranean Dietary Pattern on *N*-3 Fatty Acid Tissue Levels–A Systematic Review. Prostaglandins Leukot. Essent. Fatty Acids.

[15] Bauset C., Martínez-Aspas A., Smith-Ballester S., García-Vigara A., Monllor-Tormos A., Kadi F., Nilsson A., Cano A. (2022). Nuts and Metabolic Syndrome: Reducing the Burden of Metabolic Syndrome in Menopause. Nutrients.

[16] Cubas-Basterrechea G., Elío I., Sumalla-Cano S., Aparicio-Obregón S., González-Antón C.T., Muñoz-Cacho P. (2022). The Regular Consumption of Nuts Is Associated with a Lower Prevalence of Abdominal Obesity and Metabolic Syndrome in Older People from the North of Spain. Int. J. Environ. Res. Public. Health.

[17] Schlesinger S., Neuenschwander M., Schwedhelm C., Hoffmann G., Bechthold A., Boeing H., Schwingshackl L. (2019). Food Groups and Risk of Overweight, Obesity, and Weight Gain: A Systematic Review and Dose-Response Meta-Analysis of Prospective Studies. Adv. Nutr..

[18] Mendivil C.O. (2021). Fish Consumption: A Review of Its Effects on Metabolic and Hormonal Health. Nutr. Metab. Insights.

[19] Kim Y.-S., Xun P., Iribarren C., Van Horn L., Steffen L., Daviglus M.L., Siscovick D., Liu K., He K. (2016). Intake of Fish and Long-Chain Omega-3 Polyunsaturated Fatty Acids and Incidence of Metabolic Syndrome among American Young Adults: A 25-Year Follow-up Study. Eur. J. Nutr..

[20] Ba D.M., Gao X., Chinchilli V.M., Liao D., Richie J.P., Al-Shaar L. (2022). Red and Processed Meat Consumption and Food Insecurity Are Associated with Hypertension; Analysis of the National Health and Nutrition Examination Survey Data, 2003–2016. J. Hypertens..

[21] Oude Griep L.M., Seferidi P., Stamler J., Van Horn L., Chan Q., Tzoulaki I., Steffen L.M., Miura K., Ueshima H., Okuda N. (2016). Relation of Unprocessed, Processed Red Meat and Poultry Consumption to Blood Pressure in East Asian and Western Adults. J. Hypertens..

[22] Mendes M.I.F., Mendonça R.D.D., Aprelini C.M.D.O., Molina M.D.C.B. (2024). Consumption of Processed Meat but Not Red Meat Is Associated with the Incidence of Hypertension: ELSA-Brasil Cohort. Nutrition.

[23] Allen T.S., Bhatia H.S., Wood A.C., Momin S.R., Allison M.A. (2022). State-of-the-Art Review: Evidence on Red Meat Consumption and Hypertension Outcomes. Am. J. Hypertens..

[24] Poll B.G., Cheema M.U., Pluznick J.L. (2020). Gut Microbial Metabolites and Blood Pressure Regulation: Focus on SCFAs and TMAO. Physiology.

[25] Romaguera D., Norat T., Mouw T., May A.M., Bamia C., Slimani N., Travier N., Besson H., Luan J., Wareham N. (2009). Adherence to the Mediterranean Diet Is Associated with Lower Abdominal Adiposity in European Men and Women. J. Nutr..

[26] Hu F.B. (2002). Dietary Pattern Analysis: A New Direction in Nutritional Epidemiology. Curr. Opin. Lipidol..

[27] Huang Y., Wang S., Tian L., Zhang X., Liu S., Zhu Z., Wang W., Shi D., He M., Shang X. (2025). Healthy Lifestyle Habits, Educational Attainment, and the Risk of 45 Age-Related Health and Mortality Outcomes in the UK: A Prospective Cohort Study. J. Nutr. Health Aging.

[28] Papadaki A., Nolen-Doerr E., Mantzoros C.S. (2020). The Effect of the Mediterranean Diet on Metabolic Health: A Systematic Review and Meta-Analysis of Controlled Trials in Adults. Nutrients.

[29] Mirmiran P., Moslehi N., Mahmoudof H., Sadeghi M., Azizi F. (2015). A Longitudinal Study of Adherence to the Mediterranean Dietary Pattern and Metabolic Syndrome in a Non-Mediterranean Population. Int. J. Endocrinol. Metab..

[30] Boujelbane M.A., Ammar A., Salem A., Kerkeni M., Trabelsi K., Bouaziz B., Masmoudi L., Heydenreich J., Schallhorn C., Müller G. (2025). Regional Variations in Mediterranean Diet Adherence: A Sociodemographic and Lifestyle Analysis across Mediterranean and Non-Mediterranean Regions within the MEDIET4ALL Project. Front. Public Health.

[31] Obeid C.A., Gubbels J.S., Jaalouk D., Kremers S.P.J., Oenema A. (2022). Adherence to the Mediterranean Diet among Adults in Mediterranean Countries: A Systematic Literature Review. Eur. J. Nutr..

[32] Bajerska J., Chmurzynska A., Muzsik A., Krzyżanowska P., Mądry E., Malinowska A.M., Walkowiak J. (2019). Author Correction: Weight Loss and Metabolic Health Effects from Energy-Restricted Mediterranean and Central-European Diets in Postmenopausal Women: A Randomized Controlled Trial. Sci. Rep..

[33] Duś-Żuchowska M., Bajerska J., Krzyżanowska P., Chmurzyńska A., Miśkiewicz-Chotnicka A., Muzsik A., Walkowiak J. (2018). The Central European Diet as an Alternative to the Mediterranean Diet in Atherosclerosis Prevention in Postmenopausal Obese Women with a High Risk of Metabolic Syndrome—A Randomized Nutrition-al Trial. Acta Sci. Pol. Technol. Aliment..

[34] Hutchins-Wiese H.L., Bales C.W., Porter Starr K.N. (2022). Mediterranean Diet Scoring Systems: Understanding the Evolution and Applications for Mediterranean and Non-Mediterranean Countries. Br. J. Nutr..

